# Diminishing accelerated long-term forgetting in mild cognitive impairment: Study protocol for a prospective, double-blind, placebo-controlled, randomized controlled trial

**DOI:** 10.1016/j.conctc.2022.100989

**Published:** 2022-09-02

**Authors:** Katherine S. Adcock, Brian Lawlor, Ian H. Robertson, Sven Vanneste

**Affiliations:** Global Brain Health Institute, Institute of Neuroscience, Trinity College Dublin, Dublin, Ireland

**Keywords:** Episodic memory, Amnestic mild cognitive impairment, Mild Alzheimer's disease, Electrical stimulation, Greater occipital nerve

## Abstract

**Background:**

Harnessing the lifelong potential of the human brain for neuroplasticity may serve to maintain the viability of neural structures and postpone the onset of cognitive decline. The absence of effective pharmacological interventions to counter memory decline has encouraged scientists to test the possibility that noninvasive electrical stimulation may serve as an additional tool to improve memory abilities.

Previous research showed that electrical stimulation of the greater occipital nerve enhances memory recall performance in young and older healthy subjects. This study aims to extend these findings to determine the effect of transcutaneous electrical stimulation of the greater occipital nerve on the improvement of episodic memory in individuals with amnestic Mild Cognitive Impairment (aMCI).

**Methods/design:**

This study is a prospective, double-blind, placebo-controlled, randomized parallel-group study. A total of 100 individuals with a diagnosis of aMCI according to NIA/AA will be recruited. Participants will be randomly assigned to one of four groups. One group will receive active non-invasive transcutaneous electrical stimulation of greater occipital nerve (NITESGON), while three groups will serve as controls (i.e., sham NITESGON, active NITESGON with local anesthesia, and active NITESGON on the C5/C6 nerve). The primary outcome, i.e., memory recall, will be determined by a word association task, and will be recorded at baseline, 7 days after NITESGON, and 28 days after NITESGON. The secondary outcome is neurophysiological changes determined by resting state EEG and will be assessed immediately before and after NITESGON.

**Discussion:**

The results will add new insights into improving episodic memory in individuals with aMCI.

**Trial registration:**

#NCT05289804 (clinicaltrial.gov)

**Protocol approval id:**

#SPREC102021-23 (Ethics Committee at Trinity College Dublin, School of Psychology)

## 1 Background

Advances in medicine and public health, rising standards of living, and improvements in education and nutrition have lengthened the human life span [1], thereby increasing the prevalence of Alzheimer's disease (AD). The prevalence of amnesic mild cognitive impairment (aMCI), a prodromal stage of AD, is 10–20% in adults aged 65 years or above and increases with age [2]. Researchers posit that early identification and intervention may be the key to mitigate dementia and memory impairments. Deficits in episodic memory, including accelerated long-term forgetting (ALF), are one of the earliest detectable cognitive abnormalities in aMCI and AD [3].

ALF refers to abnormal forgetting over hours to weeks despite normal acquisition or encoding [4]. ALF is believed to be mediated by a disruption in memory consolidation, the memory processing stage in which information is transferred from short-term into long-term memory. The locus coeruleus (LC) plays a key role in driving hippocampal synaptic plasticity required for memory consolidation, in which synaptic connections are modified and strengthened to support the rapid formation of memory events [5,6]. A number of studies have documented abnormal LC activity associated with aMCI and AD [[7], [8], [9], [10], [11], [12], [13], [14]], which likely affects the hippocampal synaptic plasticity necessary for memory consolidation. Interventions that target synaptic plasticity could be promising in managing ALF by improving memory consolidation, and consequently ALF in aMCI subjects.

In a recent study we demonstrated that non-invasive transcutaneous electrical stimulation of greater occipital nerve (NITESGON) improves memory in healthy young (18–25 years) [15] and older (>65 years) [16] participants up to 28 days after stimulation. In the latter study, NITESGON was utilized in an older population to determine if stimulation during an associative memory task can optimize associative memory performance up to 28 days later. Thirty subjects between the ages of 55 and 70 years were enrolled and randomly assigned to active or sham NITESGON group. Active or sham NITESGON was delivered during the study phase at visit 1 of the associative memory task, and participants were asked to return 7-and 28-days later to measure memory recall. During visit 1, participants learned a list of 50 Swahili-English word pairs (e.g., mashua-boat) across a total of 3 blocks for each study (S) and test (T) phase. The study phase of each block consisted of 50-word pairs followed by a test phase of the 50 words, in which participants were asked to type the correct English translation for each Swahili word. Participants were then dismissed and returned for the test 7-days and 28-days later (recall phase). During this test, subjects were shown each Swahili word and were asked to type the correct English translation. This was identical to the test phase from the first visit. Participants that received active stimulation exhibited increased words recalled on days 7 and 28 compared to participants that received sham stimulation (Day 7: η^**2**^ = .21; Day28: η^**2**^ = .14). These findings suggest that NITESGON enhances memory performance up to 28-days after a single session. With these results, we propose that NITESGON could mitigate memory loss and rate of decline in the earliest stages of Alzheimer's disease.

Results also demonstrated that NITESGON promotes noradrenaline (NA) release though LC activation, followed by enhanced communication between the LC and hippocampus. NITESGON induced synaptic plasticity then modulates memory with lasting effects by strengthening memory consolidation after encoding, resulting in upregulated memory performance [15]. Furthermore, we were able to show that NITESGON induces gamma changes in the medial temporal lobe immediately after NITESGON, which was correlated with successful recall several days after stimulation. This latter finding suggest that gamma oscillations can potential be use as a biomarker for successful recall.

We seek to extend these findings to determine if NITESGON can enhance memory consolidation in aMCI subjects. To do so, we will use the word association task to assess whether NITESGON can mitigate ALF in individuals diagnosed with aMCI. In addition, we plan to assess the effect of NITESGON on EEG activity by examining the association of gamma oscillations in the medial temporal cortex with memory performance. We hypothesize that NITESGON can improve memory performance to mitigate ALF in aMCI subjects by targeting and strengthening the impaired hippocampal neuroplasticity necessary for memory consolidation. Demonstrating NITESGON induced memory improvements will be a significant step forward towards developing an effective treatment and potential early intervention for individuals with aMCI.

## 2 Method/design

### 2.1 Aims

The objective of current project is to establish the effectiveness of NITESGON in decreasing ALF in individuals with aMCI. Our primary hypothesis is that participants that receive active NITESGON while learning a word association task will perform better on word recall 7 and 28 days after NITESGON in comparison to control groups. In addition, we hypothesize that active NITESGON will significantly increase gamma oscillations within the medial temporal cortex after NITESGON in association with memory performance in individuals with aMCI 7 and 28 days after NITESGON.

### 2.2 Ethics

This study is approved by the Trinity College Data Protection Office and is in compliance with data protection legislation, specifically the EU General Data Protection Regulation 2016 (‘GDPR’), Data Protection Acts 1988–2018 and Health Research Regulations 2018. It was further approved by the Ethics Committee at Trinity College Dublin, School of Psychology (**#**SPREC102021-23).

### 2.3 Study design

This study is designed as a prospective, double-blind, placebo-controlled, randomized parallel-group study that will be completed at Trinity College Dublin, Ireland. A total of 100 individuals with amnestic mild cognitive impairment (aMCI) based on diagnostic criteria described by the National Institute on Aging and Alzheimer's Association [17] will be recruited from Alzheimer's Ireland. Subject will receive a (real or control) non-invasive transcutaneous electrical stimulation of the greater occipital nerve (NITESGON) procedure. After providing written informed consent, subjects will be assigned to one of four groups. One group will receive active NITESGON, while the three groups will be control groups. One group will receive sham NITESGON (inactive control), while a second group will receive active NITESGON with local anesthesia (active control; local nerve anesthesia group) and a third group will stimulate the C5/C6 nerve (active control; same sensation different nerve). We will include three control groups so that we are able to verify that the effect is real, location specific and cannot be associated to a sensation effect.

**Fig. 1:**
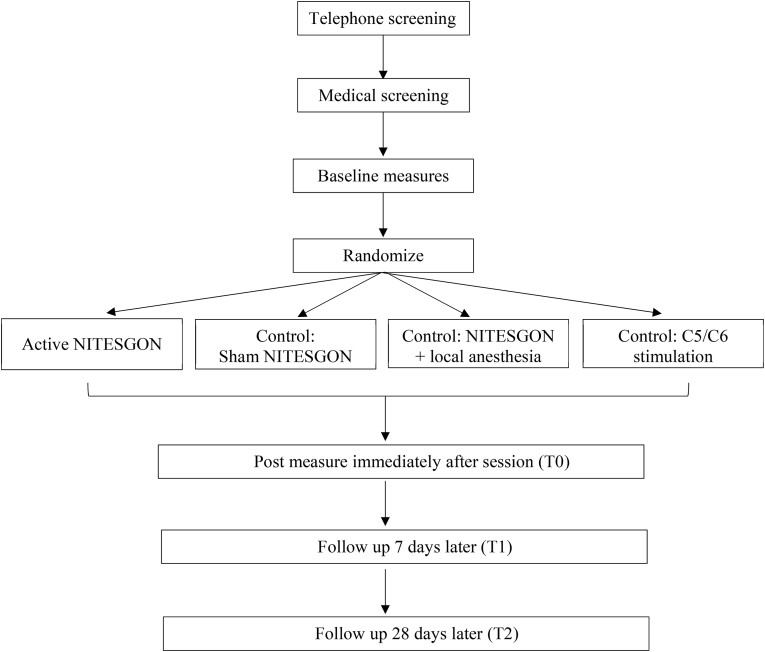
Participant flow through study.

We have opted to use a parallel-group design as it is unclear what the carry-over effect and/or wash-out period will be for NITESGON. Subjects will be randomized after screening evaluation and will be assigned to receive one of the four possible types of NITESGON. To eliminate subjective bias, all subjects and the investigator testing the endpoint measures will be blinded to the type of intervention. The researcher who will control the NITESGON device will not be involved in instructing the participant; this will be performed by a second researcher who will be blind to the stimulation protocol. The primary outcome will be change in memory performance as measured by a word association task, measured at baseline (T0), 7 days after NITESGON (T1) and after 28 (T2) days after NITESGON. Secondary outcomes include changes in resting EEG, which will be assessed immediately before and after NITESGON (see Fig. 1 and Table 1).

**Table 1:** Data collection timepoints.

	T0	T1	T2
	Baseline	7 days after NITESGON	28 days after NITESGON
Word association task	x	x	x
Neurophysiology	x		
Adverse events	x		
Blinding			x
Control assessments	x		

### 2.4 Recruitment and screening

We plan to include 100 English-speaking individuals with aMCI, recruited from Alzheimer's Ireland. Individuals will be referred to the study once a general practitioner has confirmed the diagnosis according to the Albert et al. [17] criteria. Individuals will be referred to the study nurse who will explain the study in detail and go through the eligibility criteria to see if the patient is interested in participating. If interested and eligible, the study nurse can schedule an appointment for a first visit. Additionally, patients who sought care at the memory clinic at St. James or Tallaght University Hospital for aMCI in the past will be contacted for this study if they had indicated they were interested to participate in future research studies. Advertisements will also be simultaneously implemented to recruit outside the network.

Participants will be screened and included in the study irrespective of their race. We will exclude non-English speakers because not all screening forms, questionnaires and tests are available or validated in any other language except English. To participate in this study, subjects should be able to provide an informed consent, have a confirmed aMCI diagnosis from their general practitioner and have all medications stable for at least three months prior to baseline data collection.

*Diagnostic criteria.* Participants will have a diagnosis of aMCI according to the Albert et al. [17] criteria. All enrolled participants will be classified as having memory decline based on the Clinical Dementia Rating global score of 0.5 or greater, consistent with the established classification [17,18].

*Inclusion Criteria*. One-hundred individuals, 50 years of age and above with amnestic mild cognitive impairment (aMCI) will be recruited. Participants who meet the following criteria will be eligible to participate: (1) Diagnosis of probable aMCI; (2) Montreal Cognitive Assessment between 18 and 25; (3) stable medication condition for more than 3 months; (4) stable medical condition for more than 6 months; (5) adequate visual and auditory acuity to complete neuropsychological testing; (6) capacity to provide written and dated informed consent form. Prospective participants, who meet the criteria, will be sent an electronic copy of the consent form. Participants will only be asked to sign the consent form at the site after going over the form with the researcher where all aspects of the study protocol will be reviewed.

*Exclusion criteria*. Participants will be deemed ineligible if they meet any of the following criteria: (1) any cause of cognitive decline or dementia that is not Alzheimer's disease related; (2) enrollment in any investigational drug study; (3) history in the past 2 years of epileptic seizures (participants with epilepsy who have been stable off medication or seizure free for 2 years may be included); (4) any major psychiatric disorder (a clinical diagnosis of major depressive disorder, bipolar disorder, or schizophrenia); (5) past history or MRI evidence of brain damage, including significant trauma, stroke, hydrocephalus, intellectual disability, or serious neurological disorder; (6) significant history of alcoholism or drug abuse of non-prescription drugs within the last 10 years; (7) familiarity with Swahili/Arabic language or Swahili culture due to the nature of the stimuli; (8) MCI due to vascular disease. Participants will be invited to complete MRI brain scans. Participants with pacemakers or other medical metal devices that are incompatible with MRI will not be eligible for MRI scanning as per standard procedures.

*Withdrawal of participants.* The investigator can decide to withdraw a subject from the study for urgent medical reasons. Subjects can leave the study at any time for any reason if they wish to do so without any consequences.

### 2.5 Study procedure

We will conduct this study as follows:

(1) Screening of individuals with aMCI.
(2) Randomly assigning aMCI subjects to one of the four NITESGON groups.
(3) Administering one session of 1.5-mA active (*n* = 25) or control (*n* = 25 in each of three control group) NITESGON paired with a word-association task; administered by research assistant.
(4) Behavioral assessments after each of the three blocks of studying the word associations and neural measures immediately after the last session of Behavioral assessments (T0).
(5) Behavioral assessments at seven (T1) and 28 (T2) days after NITESGON.

### 2.6 Intervention

NITESGON will be delivered by a specially developed, battery-driven, constant current stimulator with a maximum output of 10 mA (http://www.neuroconn.de) via a pair of saline-soaked surface sponges (35 cm^2^) on the scalp. One electrode will be placed over the left and right C2 dermatomes each. A constant current of 1.5 mA intensity will be applied during each of the 3-study phases (i.e., 250 s x 3 blocks). For sham NITESGON, placement of the electrodes will be identical to active NITESGON. A third group (local nerve anesthesia group) will receive NITESGON, but we will use a topical skin anesthetic (lidocaine/prilocaine cream) to reduce any potential contribution from transcutaneous stimulation of peripheral nerves. This lidocaine/prilocaine preparation blocks sodium channels in peripheral nerves in the skin and thereby stabilizes the membrane potential and increases the threshold for firing an action potential [15,19]. For the fourth control group (different nerve group), the electrodes will be placed over cervical nerves five and six, to mimic the sensation, but change the location. For both the third and fourth control group, we will apply the same current as active NITESGON. NITESGON will first be switched on in a ramp-up fashion over 30 s. For sham, the current intensity (ramp down) will gradually be reduced (over 5 s) as soon as NITESGON reaches a current flow of 1.5 mA. The rationale behind this sham procedure is to mimic the transient skin sensation at the beginning of active NITESGON without producing any conditioning effects on the brain.

### 2.7 Outcome assessment

A variety of measures are included at the T0, T1, and T2 evaluations. The primary outcome measure is improvement in memory recall. Recall will be assessed using the word-association task during the learning phase at visit one (T0), 7 (T1) and 28 (T2) days after learning during NITESGON.

#### 2.7.1 Primary outcome measures

*Word-association task.* Subjects will perform a Swahili–English word association task, based on a well-known paradigm [20]. Subjects will be asked to learn a list of 50 Swahili–English word pairs (e.g. mashua–boat) selected from previously published norms [20]. Subjects will learn the list of word pairs across a total of 3 blocks, in which each block consists of a study (S) and test (T) phase. The study phase of each block will consist of 50-word pairs followed by a test phase of the 50 words. During study trials, subjects will see each Swahili word and its English translation on a computer screen simultaneously for 5 s and will be told to study the word pairs so that they can recall the English word when given the Swahili word. After every study period, there will be a 30-s rest period (consolidation). During the test trials, subjects will be presented with the Swahili word and asked to type the correct English translation. Each test trial will last 16 s, after which the computer program will automatically advance to the next item regardless of whether the patient has entered a response. If subjects fail to recall an item during testing, they will not be given any feedback. In the following Blocks 2 and 3, subjects will study the entire list in each study phase but only items that they had not yet recalled in the previous block will be tested in the test phase. Subjects will then be dismissed and asked to return for a follow-up test 7 days and 28 days later (recall phase). During this test, subjects will be shown each Swahili word for 16 s and asked to type the correct English translation. This is identical to the test phase from the learning period.

#### 2.7.2 Secondary outcome measures

(1) Neurophysiology. Rest-state EEG will be collected immediately before and after NITESGON (T0) and will be correlated with the recall of the word-association task 7 (T1) and 28 (T2) days after NITESGON.
(2) TES Adverse events*.* We will use the NITESGON exit questionnaire developed by Brunoni et al. [21] to assess potential side-effect (headache, neck pain, scalp pain, tingling, itching, burning sensation, skin redness, sleepiness, trouble concentrating, mood changes) at the end of T0.
(3) Blinding. At the end of the experiment (T2), subjects will be asked if they thought they were assigned to the control or active group.
(4) Control assessments. We will collect the California Verbal Learning Test II, Delis Kaplan Color Word Interference Test, Beck Depression (BDI) and Anxiety Inventory (BAI), Trial A & B, COWAT and DIGIT Span to verify that the effect obtained cannot be associated due to differences between groups on these control measures. These will be collected during visit 1 before NITESGON (T0), and 28 days after NITESGON (T2).

### 2.8 Safety

Transcutaneous electrical stimulation (tES) is a method that has been used in various clinical populations, including pain, Parkinson's disease, movement disorders, motor strike, aphasia, multiple sclerosis, epilepsy, disorders of consciousness, Alzheimer's disease, tinnitus, depression, schizophrenia, substance abuse, addiction, and craving [22]. Based on a previous review, there is currently no evidence of irreversible injury produced by conventional tES protocols using a wide range of stimulation parameters (≤40 min, ≤ 4 mA, ≤ 7.2 C) [23]. Furthermore, most adverse effects are mild and disappear soon after stimulation [24]. Another paper that summarizes 567 tES sessions over motor and non-motor cortical areas (occipital, temporal, parietal) revealed that according to the present safety guidelines, tES is associated with relatively minor adverse effects (including headache, itching or tingling sensation) in healthy humans and individuals with various neurological disorders [25]. To date, no significant differences in side effects have been observed after receiving active NITESGON compared to sham NITESGON [15,16,26].

### 2.9 Sample size

The study is designed to give us adequate power to detect clinically meaningful differences between the groups. We plan to oversample to a target sample size of 25 subjects per group, with an overall sample of 100 subjects, to assure that our power to detect effects is maintained.

Nineteen individuals per group (total n = 76) would offer us a power of 90% to detect the difference between groups using Cohen's d estimation of a medium effect size (0.20). A sample size of 76 would allow us to see a medium effect size, however the trial is longitudinal which could increase its drop-out rate. Although we do not expect attrition to be large, we expect a dropout rate of no more than 20%. Therefore, an overall sample of 100 subjects will assure enough power to accommodate for the expected drop-out rate.

An intention-to-treat and per-protocol analysis will be performed. For the intention-to-treat analysis, the patient must successfully complete T0 and T1 assessments. For missing observations, the LOCF approach will be used. Exclusion from the population will be finalized prior to database lock in a blinded manner.

### 2.10 Statistical analysis

For our calculations, we will use the normal distribution assumption, which we will test using a normal plot and goodness of fit test. If violations are found, non-parametric tests or transformations will be utilized. Variance equivalence will be tested using the F-test. Our protocol is designed to assure sample independence, and adherence will be closely monitored. Before beginning the main statistical analyses, screening variable levels (e.g., age, sex, education, disease onset, mood) across groups will first be considered to assess group equivalence. If the groups are not balanced on any baseline characteristics, these factors will be considered as co-variate in later multivariate regressions.

The efficacy of NITESGON will be assessed on word recall by testing its effect on the mean change over time relative to the control groups. Memory task performance will be analyzed over time at baseline, and 7 and 28 days after NITESGON, and across groups. Statistically, these tests will be single degree-of-freedom interaction contrasts from a general mixed linear model containing both within- and between-subject variance components. All tests will be based on directional hypotheses in favor of improvements due to NITESGON on the behavioral assessment. We expect large behavioral effects based on our previous studies in control and patient populations.

*EEG.* sLORETA statistical contrast maps between pre and post NITESGON will be calculated for the sham and active groups through multiple voxel-by-voxel comparisons in a logarithm of F-ratio [27]. Statistical analysis is based on estimating, via randomization, the empirical probability distribution for the max-statistic under the null hypothesis comparisons [28]. This methodology corrects for multiple testing (i.e., for the collection of tests performed for all voxels and for all frequency bands). Due to the non-parametric nature of the method, its validity does not rely on any assumption of Gaussianity [28]. A comparison will be made between the contrast map for the active and sham group. The significance threshold is based on a permutation test with 5000 permutations. In addition, the statistical contrast map (pre-post) will be correlated with the difference on in memory improvement.

*TES Adverse events.* A comparison will be conducted between the active and sham group using a one-way ANOVA with stimulation (active NITESGON vs sham NITESGON) as independent variable and adverse events as dependent variable.

*Blinding.* A Chi-square test will be conducted comparing actual stimulation (active NITESGON vs sham NITESGON) versus participants' expected stimulation (active NITESGON vs sham NITESGON).

### 2.11 Data management

All data will be managed using unique study codes, which will be used to code and file all electronic information, to protect participant confidentiality. The key linking these codes to participants' identities will be stored in a secured file. Access to this key will be available only to designated members of the research team at each site. To ensure privacy, all research files will be stored in locked file cabinets in locked offices at Trinity. Electronic information will be stored at a secure, password-protected, server at Trinity and all corresponding data analysis will be conducted at Trinity to ensure central and complete data protection. Subject names will not be published. Summary statistics will be performed on all variables and reported.

## 3 Discussion

ALF is an early pre-symptomatic feature of autosomal dominant Alzheimer's disease, which appear to pre-date other amnestic deficits and underpin subjective memory complaints in AD [29]. Walsh and colleagues found that aMCI subjects showed greater loss of information over 1 week than controls and that delayed memory recall deficits in aMCI subjects appeared to be related to impaired consolidation when controlling for the initial learning [30]. Similarly, we predict that individuals with aMCI will perform significantly worse 7 days after initial learning of the word association task. The objective of the current project is to determine if NITESGON is an effective intervention to improve memory recall and therefore mitigate ALF in individuals with aMCI.

To the best of our knowledge, there have been no trials evaluating the effect of electrical stimulation of the greater occipital nerve on episodic memory in aMCI populations. LC degeneration has been associated with aMCI [7,[10], [11], [12], [13], [14]], with LC neuropathology detectable as early as 10 years before neurocognitive signs [[31], [32], [33]]. Furthermore, NITESGON can upregulate NA through the LC-NA pathway. Therefore, this approach could potentially benefit individuals with aMCI. The secondary outcome measures will examine mechanisms that may underlie the memory effect these populations.

Early identification of disease combined with effective intervention is the optimal strategy to alleviate memory impairments in aMCI subjects and ultimately maintain quality of life. NITESGON has the potential to improve memory recall by mitigating the disruption of memory consolidation in individuals with aMCI. This new memory management system is non-invasive, non-pharmacological, safe, with few adverse side effects, portable and can be readily applied at a reasonable cost. In addition, NITESGON can be implemented in individuals on a variety of different medications that have not been responsive to other treatments.

## Author contributions

All authors made a significant contribution to the work. SV, BL, and IHR conceived and designed the experiments. KSA and SV wrote the manuscript.

## Declaration of competing interest

The authors declare that they have no known competing financial interests or personal relationships that could have appeared to influence the work reported in this paper.
